# Fitness and Performance Testing of Male and Female Beach Soccer Players—A Preliminary Investigation

**DOI:** 10.3389/fspor.2021.636308

**Published:** 2021-02-23

**Authors:** Malte N. Larsen, Georgios Ermidis, João Brito, Cecilie Ørner, Clarice Martins, Luís Filipe Lemos, Peter Krustrup, Vincenzo Rago

**Affiliations:** ^1^Department of Sports Science and Clinical Biomechanics, Faculty of Health Science, University of Southern Denmark, Odense, Denmark; ^2^Department of Sports and Well-Being “Parthenope” University of Naples, Naples, Italy; ^3^Portugal Football School, Portuguese Football Federation, Oeiras, Portugal; ^4^Research Center in Physical Activity, Health and Leisure, Oporto University, Porto, Portugal; ^5^Federal University of Paraíba, João Pessoa, Brazil; ^6^Faculty of Health Sciences and Sports, Universidade Europeia, Lisbon, Portugal

**Keywords:** fitness, performance, Beach soccer, sprint, power

## Abstract

**Purpose:** This study aimed to compare performance on sand and a firm surface and to describe the physical capacity of male and female beach soccer players.

**Methods:** Sixty-six male and 29 female competitive beach soccer players voluntarily participated in this study. Firstly, within-subjects test scores were compared to scores on a firm surface (criterion validity; *n* = 15 men) and reconducted on a second occasion (reliability; *n* = 51 men). Secondly, the best score on sand was retained to compare test performance between ages (classified as below 20, 20–30, and above 30 years) and sexes. Performance assessments included sprint time over 5 and 15 m (once on a firm surface and twice on sand), standing long jump (SLJ, once on a firm surface and twice on sand) and Yo-Yo intermittent recovery level 1 (Yo-Yo IR1, once on a firm surface and once on sand; only data for men were available).

**Results:** Five-m sprint and Yo-Yo IR1 performance on sand were not correlated to performance on a firm surface (*P* > 0.05). Test-retest reliability was acceptable for the 15-m sprint and SLJ tests (ICC > 0.90; CV < 5%). Performance in 15-m sprint and maximal sprinting speed were moderately lower in male players aged above 30 years. compared to players aged below 30 years (*d* = 0.35–0.42; *P* < 0.05). Irrespective of the age group, weight-bearing power-based performance mass was moderately to very largely higher in male players than in female players (*d* = 0.42–0.88; *P* < 0.05).

**Conclusions:** The lack of a consistent relationship between performance on sand and on a firm surface might indicate the need to develop specific test batteries for sand-based athletes. Age-related differences in physical performance were evident only in sprint capacity. Further studies are warranted to elucidate our preliminary findings and to develop the sand specific tests.

## Background

Beach soccer is a relatively new sport, having its beginning in the 1990s, and currently one of the fastest developing sports worldwide (FIFA). More than 50 countries worldwide play beach soccer, including 24 countries in Europe (Dansk Bold Union Beach soccer, 2019).

Beach soccer is characterized as an intermittent sport of high intensity involving specific actions such as accelerations, jumps, and passes with the added difficulty of executing these skills on an unstable surface (sand). This places high demands on energy and the anaerobic system, with the average intensity >90% of maximum heart rate during most of the match (Rosario et al., 2015; Scarfone and Ammendolia, 2017; Leite, 2021). This can be related to the small size of the beach soccer pitch (36.5 × 27.5 m) and the possibility of free substitutions. A team consists of more than twice as many players as the five players allowed on the pitch at the same time, which increases the demands on the players' anaerobic capacity and power, and the importance of fast recovery.

Despite the increasing popularity of beach soccer worldwide, played either at amateur or professional level, there is little research on this sport, with only one study describing the characteristics of beach soccer players (Leite, 2021) and independent of the specific sand sport soccer, there is a regular practice of training at sand surfaces from soccer teams, where specific measurements and test could be developed. This study aimed to, compare performance on sand and a firm surface and to describe physical capacity by age and sex in Brazilian beach soccer players at regional championship level.

## Materials and Methods

A total of 95 competitive beach soccer players (66 males, 29 females) voluntarily participated in this study. The participants were involved in specific beach soccer training at least twice a week (on average 90 min per session) and 1–2 physical/strength sessions per week involving plyometrics, injury prevention, and power training and they were tested during the competition period in March 2019. Firstly, within-subjects test scores were compared to scores on a firm surface (criterion validity; *n* = 15 men) and reconducted on a second occasion (reliability; *n* = 51 men). Secondly, the best score on sand was retained to compare test performance between ages (classified as below 20, 20–30, and above 30 years) and sexes. Performance assessments included sprint time over 5 and 15 m (once on a firm surface and twice on sand), standing long jump (SLJ, once on a firm surface and twice on sand), and Yo-Yo intermittent recovery level 1 [Yo-Yo IR1, once on a firm surface and once on sand (only data for men were available)]. Assessment on sand and a firm surface was performed on separate days, minimum 2 days and maximum 14 days apart. During the 4-days tournament, information about environmental conditions was recorded via the Brazilian Government's Center for Weather Forecasts and Climate Studies. Temperature ranged between 26.8 and 30.6°C, air humidity between 64 and 70% and wind velocity between 2.44 and 3.10 m/s.

Sample characteristics can be found in Table 1.

**Table 1:** Sample characteristics.

			**Height (cm)**		**Body mass (kg)**	
		***N***	**Mean**	**SD**	**Mean**	**SD**
Age < 20 years	Male	16	172.4	6.5	65.8	11.0
	Female	10	161.4	6.1	54.8	4.0
Age 20–30 years	Male	32	174.9	4.9	72.4	9.2
	Female	19	160.6	6.3	60.3	7.2
Age > 30 years	Male	18	173.6	6.6	76.6	14.1
	Female	0				
Total	Male	66	173.9	5.8	71.9	11.0
	Female	29	160.9	6.2	58.4	6.1

### Anthropometrics

The anthropometric variables of height (m) and body mass (kg) were recorded from each subject. Height was measured using a stadiometer (Holtain Ltd., Pembrokeshire, UK), and body mass was measured with a bioimpedance scale (InBody 570, Biospace Co. Ltd., Seoul, Korea).

### Jump Performance

Standing long jump (countermovement) performance was measured after a warm-up programme. The participants performed two jumps separated by a 5–10-min rest. The jumps were performed wearing sports shoes or with bare feet. The participants stood still with their feet parallel and shoulder-width apart, with toes just behind a line. They were instructed to bend their knees to a 90-degree squat position before jumping as far as possible using their arms to generate power, researchers controlled the players by subjective evaluation, and told them to redo the jump, if the instructions wasn't followed close enough. The distance from the start line to the back heel on landing was measured in centimeters. Each player had two tries 5–10 min apart, and the longest jump was reported as the result. The standing long jump is a valid test strongly associated with upper- and lower-body maximal muscle strength and showing moderate-to high reliability (Markovic et al., 2004).

### 0–5-m Acceleration and 0–15-m Sprint

Acceleration and sprint times were measured using photocells (Speed Test 6.0 standard, Cefise, São Paulo, Brazil). The participants performed two 15-m sprints, one on sand and one on a firm surface, separated by 5 min of rest. The starting position was standardized, with the lead-off foot behind the starting line, which was located 1-m behind the first time gate. The photocell gates were placed at the start, and at 5 and 15 m. The subjects attempted to cover the 15 m as quickly as possible. The best time from the two attempts was recorded (0–5 m acceleration; 0–15 m sprint). When the test was performed on sand, the conditions were standardized. After each attempt, the sand was smoothed with a squeegee, ensuring the same conditions for all athletes in all attempts.

### Cardiovascular Fitness

Running performance was evaluated by Yo-Yo IR1. The test was performed once on a firm surface and once on sand. The test consisted of two 20-m shuttle runs at progressively increasing speeds, separated by 10 s of jogging after each bout of running, around a cone placed 5 m behind the start line. Each run was separated by a beep played through loudspeakers. The frequency of the beeps increased during the course of the test. The first time the participant failed to reach the finish line in time, a warning was given; the second time, the test ended. Total running distance was recorded. Yo-Yo IR1 is a valid test strongly associated with aerobic fitness levels (Schmitz et al., 2018).

The tests were conducted within the season at a time when the players were supposed to be in their best condition.

A mixed linear model was used to assess age- and sex-related differences, while a paired sample *t-*test was used to assess surface-related differences in performance and test-retest reliability. The *t* statistics derived from either the mixed model or *t*-test were used to calculate the magnitude of differences (*d*) (Rosnow et al., 2000). Correlations between performance on a firm surface and on sand, and between test and retest, were evaluated using Pearson's product moment (Hopkins et al., 2009).

## Results

Five-m sprint and Yo-Yo IR1 performance on sand were not correlated to performance on a firm surface (*P* > 0.05), with correlation coefficients at *r* = 0.14 and *r* = 0.34, whereas 15-m sprint and SLJ performance on sand and firm service was correlated (*r* = 0.79 and *r* = 0.61, irrespectively; *P* < 0.05). The results can be found in Table 2.

**Table 2:** Validity of sand-based performance tests vs. a firm surface (only data for men available, *n* = 15).

	**Firm**		**Sand**		**Correlation**				**Difference**			
	**Mean**	**SD**	**Mean**	**SD**	**r**	**95LB**	**95UB**	***P***	**d**	**95LB**	**95UB**	**P**
5-m sprint time (s)	1.02	0.04	1.06	0.08	−0.14	−0.74	0.58	0.715	0.42	−0.41	0.87	0.228
15-m sprint time (s)	2.45	0.08	2.53	0.12	0.79	0.26	0.95	0.011	0.73	0.05	0.95	0.017
SLJ (m)	2.16	0.18	1.80	0.90	0.61	0.02	0.89	0.046	0.42	−0.28	0.83	0.169
Yo-Yo IR1 (m)	816	236	597	162	0.34	−0.21	0.73	0.216	0.69	0.25	0.89	0.003
HR_max_ (bpm)	188.3	8.8	190.0	6.8	0.51	0.00	0.81	0.050	0.22	−0.35	0.67	0.411

*HR_max_, maximum heart rate; SLJ, standing long jump; Yo-Yo R1, Yo-Yo intermittent recovery test level 1*.

Test-retest reliability was acceptable for the 15-m sprint and SLJ tests (ICC > 0.90), while it was only found to be moderate for 5-m sprint time (ICC = 0.74). The results can be found in Table 3.

**Table 3:** Reliability of sand-based performance tests (only data for men available, *n* = 51).

	**Firm surface**	**Test**		**Retest**		**Test-retest correlation**					**Test-retest variation**		
	***N***	**Mean**	**SD**	**Mean**	**SD**	**ICC**	**95%LB**	**95%UB**	**Inference**	**P**	**CV (%)**	**Inference**	**SWC**
5-m sprint time (s)	51	1.09	0.09	1.07	0.08	0.74	0.58	0.86	Moderate	<0.01	1.30	Good	0.39
15-m sprint time (s)	51	2.57	0.13	2.54	0.13	0.90	0.83	0.94	Good	<0.01	0.83	Good	0.25
SLJ (m)	50	2.13	0.17	2.16	0.19	0.96	0.93	0.97	Good	<0.01	0.98	Good	0.30
Yo-Yo IR1 (m)	No re-test												
HR_max_ (bpm)	No re-test												

Performance in 15-m sprint and maximal sprinting speed were moderately lower (4–5%) in male players aged above 30 compared to players aged below 30 (*d* = 0.35–0.42; *P* < 0.05). The results can be found in Table 4.

**Table 4:** Performance of beach soccer players by age group and sex.

**Performance on sand**		**Below 20 years**			**20–30 years**			**Above 30 years**		
		**Mean**	**95LB**	**95UB**	**Mean**	**95LB**	**95UB**	**Mean**	**95LB**	**95UB**
5-m sprint time (s)	Male	1.01	0.97	1.05	1.02	0.99	1.05	1.07	1.03	1.11
	Female	1.11	1.08	1.15	1.11	1.06	1.16			
15-m sprint time (s)	Male	2.45	2.39	2.51	2.47	2.428	2.52	**2.58**	**2.52**	2.64^*^#
	Female	2.83	2.76	2.89	2.81	2.71	2.91			
Maximal sprinting speed (km/h)	Male	24.86	24.28	25.44	24.91	24.49	25.34	23.87	23.29	24.45^*^
	Female	21.03	20.49	21.57	21.15	20.37	21.94			
Standing long jump (m)	Male	2.07	1.73	2.41	2.089	1.85	2.329	1.964	1.644	2.283
	Female	1.51	1.34	1.68	1.69	1.454	1.926			
Yo-Yo IR1 (m)	Male	695	533.3	856.7	584	439.4	728.6	520	62.6	977.36
	Female									

* *Significantly different from players aged below 20 years (P < 0.05)*.

# *Significantly different from players aged 20–30 years (P < 0.05)*.

Irrespective of the age group, weight-bearing power-based performance was moderately to very largely higher in men than in women (*d* = 0.42–0.88; *P* < 0.05). The results can be found in Table 5.

**Table 5:** Differences in power-based performance by sex.

**Performance variable**	**Age**	**Mean difference (95%CIs)**			**df**	***P***	***r***	**95LB**	**95UB**	**Inference**
Sprint time, 5-m (s)	Below 20	−0.106	−0.129	−0.083	31	0.000	0.64	0.37	0.81	Large, favors men
	20–30	−0.088	−0.12	−0.056	36	0.009	0.42	0.10	0.66	Moderate, favors men
Sprint time, 15–m (s)	Below 20	−0.374	−0.418	−0.33	31	0.000	0.84	0.69	0.92	Very large, favors men
	20–30	−0.342	−0.394	−0.29	36	0.000	0.74	0.54	0.86	Large, favors men
Standing long jump (m)	Below 20	0.585	0.519	0.651	27	0.000	0.86	0.72	0.94	Very large, favors men
	20–30	0.538	0.474	0.602	36	0.000	0.81	0.66	0.90	Very large, favors men
Maximal sprinting speed (km/h)	Below 20	3.832	3.463	4.201	31	0.000	0.88	0.77	0.94	Very large, favors men
	20–30	3.758	3.261	4.255	36	0.000	0.78	0.61	0.88	Very large, favors men

## Discussion

The main finding of the present study was the lack of a consistent relationship between performance on sand and on a firm surface, which indicates the need to develop specific test batteries for sand-based athletes. Since maximal muscle strength and aerobic cardiovascular capacity are important for performance in beach soccer (Rosario et al., 2015; Scarfone and Ammendolia, 2017; Leite, 2021), this challenges previous findings suggesting that land-based tests can be used as relevant evaluation of beach volleyball players (Bishop, 2003). Furthermore, beach soccer itself seems to be effective as aerobic fitness training for elite and recreational players (Yong, 2009; Brito et al., 2012), so a field test to evaluate the effects in both elite and recreational players would be of great relevance and might challenge the previous findings that aerobic power in beach soccer players and regular soccer players are similar (Miri et al., 2012), which is also seen when comparing the performance in the present study to that in studies examining male and female elite soccer players (Rodríguez-Fernández et al., 2018; Castagna et al., 2020). However, the biomechanics of jumping in sand are different compared to jumping on a firm surface (Giatsis et al., 2004; Riggs and Sheppard, 2009). Considering that lower values on sand have been attributed to a lower reuse of elastic energy and foot slippage (Binnie et al., 2014), it will be more relevant to have valid and reliable sand-based performance tests for beach soccer players. This is highlighted by the fact that that elite beach volleyball players have relatively strong legs when compared with studies assessing athletes using isokinetic devices at the same testing speeds, but lower vertical jump performance when compared to previous studies of elite indoor volleyball players (Davies, 2002; de Lira et al., 2017).

Regarding sprint performance, previous studies have found that in sand, the energy cost of a low-speed running is 24% higher than in a firm surface (Zamparo et al., 1992).

This percentage decreases for maximal speeds. Considering than in a 5-m acceleration test the speed is submaximal, and increases for the 15 m sprint, it is plausible that we may see a correlation for longer sprints, which also occur in beach soccer (Leite, 2021). With regards to the Yo-Yo test, its intermittent characteristic, combined with change of direction, might explain the lack of correlation.

The findings in the present study relating to maximal muscle strength might also be of importance in terms of lowering injury risk and thereby ensuring that the best players are available for all matches (Shimakawa et al., 2016; Sharifatpour et al., 2020), since injuries are found to be frequent in beach soccer (1,541 incidents per 1,000 player hours) (Porramatikul et al., 2018).

Age-related differences in physical performance were evident only in sprint capacity. This area of research is totally new in beach soccer, but has been described in regular soccer, where professional soccer players aged >30 years showed significant lower performance in total distance covered, number of fast runs and number of sprints compared with younger players (≤30 years) (Rey et al., 2019). On the other hand, the players' ability to make successful passes increased with age (Sal de Rellán-Guerra et al., 2019). Understanding age-related attributes can help team managers to design their rosters optimally and/or focus on physical training and rest, especially for older players, since the variation in their performance is also greater than seen in younger players (Lorenzo-Martínez et al., 2020).

### Strengths and Limitations

The strength of the study is the level and number of participating players, providing knowledge of elite players, close to world-class in both genders, whereas the limitation is the number of variables measured, since more variables (i.e., from muscle or blood samples) describing aerobic, anaerobic and strength-related performance could be useful, as well as even more participants for subgroup analysis divided by different playing position, which has been shown to be important in terms of physiological match demands in soccer in both genders (Bradley et al., 2014; Datson et al., 2017).

## Conclusions

The lack of a consistent relationship between performance on sand and on a firm surface might indicate the need to develop specific test batteries for sand-based athletes. Age-related differences in physical performance were evident only in sprint capacity, whereas gender differences was found in all evaluated parameters. Further studies are warranted to elucidate our preliminary findings.

## Data Availability Statement

The raw data supporting the conclusions of this article will be made available by the authors, without undue reservation.

## Ethics Statement

The studies involving human participants were reviewed and approved by The evaluation methods and procedures were approved by the local Ethics Committee (University Center of João Pessoa, Paraíba) with protocol number 19016819.2.0000.5176. Written informed consent for participation was not required for this study in accordance with the national legislation and the institutional requirements.

## Author Contributions

ML participated in designing the study, data collection, analysis, and finalized the first draft of the manuscript. GE contributed to the data analysis and the final draft of the paper. JB and PK contributed to the design and the final draft of the paper. CØ has worked on the first draft of the paper. CM and LL participated in designing the study, data collection, and the final draft of the paper. VR and GE contributed to the data analysis and the final draft of the paper. All authors contributed to the article and approved the submitted version.

## Conflict of Interest

The authors declare that the research was conducted in the absence of any commercial or financial relationships that could be construed as a potential conflict of interest.
